# Assessment of Self-Care in Promoting Healthy Aging Among the Elderly in Rural Areas of Kancheepuram, Tamil Nadu

**DOI:** 10.7759/cureus.84171

**Published:** 2025-05-15

**Authors:** Swathika Devi R, Anantha Eashwar V. M., Sujitha Pandian, Monica Albert Sekhar

**Affiliations:** 1 Community Medicine, Sree Balaji Medical College and Hospital, Chennai, IND; 2 Preventive Medicine, Sree Balaji Medical College and Hospital, Chennai, IND

**Keywords:** activities of daily living, barthel index, functional ability, geriatric population, health management, well-being

## Abstract

Background

India's elderly population is rapidly growing, facing various social and economic challenges, with nearly half suffering from chronic diseases affecting their quality of life. Self-care, as defined by the World Health Organisation, plays a crucial role in maintaining health, independence, and life satisfaction among the elderly, though it is influenced by factors like socio-economic status and social support. With the goal to promote healthier aging, this study aims to assess the prevalence of self-care among the elderly aged 60 years and above and also to determine the factors influencing their self-care practices.

Methods

This cross-sectional study was conducted in the rural field-practice areas of a private medical college and hospital, Kancheepuram, among 225 elderly aged 60 years and above. The study participants were selected through simple random sampling. After obtaining informed consent, data collection was carried out using a pre-structured and pre-tested questionnaire. Data was entered into MS Excel (Microsoft® Corp., Redmond, USA) and analyzed using IBM SPSS Statistics for Windows, Version 25 (Released 2017; IBM Corp., Armonk, United States). Descriptive statistics were presented in tables, and analytical statistics, including the chi-square test, were performed to determine associations between related variables.

Results

Among the 225 participants, the majority of the people belonged to the age group of 60 to <75 years, 74.2% were married, and around 51% were financially supported by their families. Poor self-care was seen among 38% of the participants. Poor self-care was seen to be higher among the elderly >75 years of age. Participants with an illness duration of more than 5 years, those who did not take medications on their own, and those who were not on routine follow-up, were found to have statistically significant factors of poor self-care.

Conclusion

Self-care is essential since elderly people regularly deal with limitations and concerns that are often disregarded. It is crucial to teach healthcare professionals self-care techniques so that older people, especially those with non-communicable diseases, are aware of them. Families and caregivers are also essential in promoting the health of the elderly and ought to get education on self-care practices

## Introduction

Population ageing is a global phenomenon with a shift in the country’s population towards older ages. According to the World Population Prospects 2022, one in six people will be aged 60 years and older by 2030. The world’s population of people aged 60 years and above will double by 2050, and people aged 80 years or older are expected to triple between 2020 and 2050 [1].

According to the 2024 World Population Data Sheet, it is expected that a growing elderly population will bring an increase in chronic diseases such as hypertension, diabetes, and cardiovascular diseases, and there will be an increase in the need for caregiving. Preventive measures to maintain the health of the elderly and delay the onset of age-related diseases help reduce the long-term burden on the health system [2,3] 

India’s elderly population is expected to increase from 138 million in 2021 to 193.4 million by 2031, as per the Report of the Technical Group on Population Projections (July 2020) [4,5]. This rapid growth in the elderly population can be attributed to economic well-being, reduction in fertility rates, and improvement in healthcare and medical services and facilities. Senior citizens face many social and economic challenges, such as isolation, social exclusion, income insecurity, financial dependency, etc. Inadequate housing and public places that are not elderly-friendly also hinder their accessibility. Nearly 50% of the elderly suffer from chronic diseases, and 5% have difficulty in physical mobility [6]. Chronic diseases and functional ability influence the quality of life of the elderly, and the risk of multiple comorbidities may lead to an increase in disability [7].

A major aspect of supporting the aging population is to ensure their ability to engage in self-care. WHO defines self-care as the ability of individuals, families, and communities to promote and maintain their own health, prevent disease, and cope with illness, with or without the support of a caregiver [8]. Self-care is directly related to their quality of life, mental health, and day-to-day activities. It includes activities that we do for survival, healthy functioning, continuous improvement, and feeling well. Self-care helps the elderly to better manage their health and to stay independent [9]. Basic personal care, such as grooming, bathing, dressing, and health-promoting behaviours such as eating a balanced diet, regular physical activity, taking medications on time, and maintaining mental well-being, comprises self-care.

With advancing age, people need more time to heal and recover from illnesses. It affects their abilities and motivation to take care of themselves. Self-care in older people is related to many factors, including level of education, socio-economic status, and social support. Any adverse life events decrease the energy for self-care [9-11]. Special attention is needed for people with low socioeconomic levels and minorities [12]. Self-care should be considered a routine and must be seen as an integral part of health care. Adherence to medication, disease management, and avoiding risky behaviours are attributed to better self-care [13].

Elderly self-care is associated with life satisfaction, self-esteem, functional capability, family support, and education [10,11]. Self-care practices maintain the autonomy of the individual and delay dependency on others for activities of daily living. Old age does not necessarily mean disability. Self-care programs should be provided for the elderly to control chronic illnesses and delay disability. Promoting self-care among the elderly and creating awareness about the key effects of self-care on healthy ageing is crucial now and a necessity of health care [9]. Therefore, this study aims to estimate the prevalence of self-care among individuals aged 60 years and above and to determine the factors associated with self-care practices of the elderly.

## Materials and methods

Study design

The present study was a community-based cross‐sectional study conducted among the elderly aged 60 years and above in the rural field-practice areas of a private medical college and hospital in Kancheepuram, India, between February 2024 and July 2024. The study included elderly aged 60 years and above residing in the rural areas of Kancheepuram after receiving informed consent from them. Those who could not provide the necessary information, i.e., people who were mentally unstable and who were not present at the registered address, were excluded from the study. Ethical clearance for the study was obtained from the Institutional Human Ethics Committee (IHEC) of Sree Balaji Medical College and Hospital, under reference number 002/SBMCH/IHEC/2023/2098.

Sample size

Based on the study by Shubanshu Gupta et al. [14], the prevalence of self-care practices among the elderly was found to be 23.4%. The sample size was calculated as follows: p=23.4%, q=76.6%, d=absolute precision of 6%,

$n = \frac{Z^2 \cdot p \cdot q}{d^2}$

$n = \frac{(1.96)^2 \times 23.4 \times 76.6}{6^2}$

$n = 191.6$

Where p = estimated prevalence of the condition (in percentage), q = 100−p (i.e., the proportion of individuals not having the condition), and d = absolute precision (acceptable margin of error in percentage).

Adding a 10% non-responsive rate, n = 211, rounded off to 225.

Sampling method

The total population of the area of the rural health training center of a private medical college and hospital was 45130. Among them, the population of the elderly aged 60 and above was 4738. With the aid of the register available at the rural health center of the college, a simple random sampling was done using the lottery method. Each elderly individual was assigned a unique number, and numbers were drawn randomly until the required sample size was attained. If the participant with the chosen unique number was not available due to any reason, the consecutive number was considered.

Study tools

A modified pre-tested semi-structured questionnaire, which includes Barthel’s index for assessing self-care, was used for data collection (see Appendices). Barthel’s index is a 10-item questionnaire that measures functional independence in activities of daily living (ADL) [14]. The total score ranges from 0 to 100, indicating the level of dependence or independence of an individual. A score between 0 and 20 reflects total dependence, scores ranging from 21 to 60 represent severe dependence, scores between 61 and 90 denote moderate dependence, scores from 91 to 99 suggest slight dependence, and a score of 100 signifies total independence. A study done by Austen El Costa et al states that, a higher score on the Barthel index indicates a higher level of self-care ability [15]. Hence, in our study, the Barthel index with a score between 91 and 100 is considered indicative of good self-care, whereas a score of 90 or below is indicative of poor self-care.

Statistical analysis

Data collected was entered into Microsoft Excel (Microsoft Corporation, Redmond, USA), and the data analysis was done using Statistical Package for Social Sciences software version 25 (Released 2017; IBM Corp., Armonk, United States). The association between variables was analysed using chi-square test and Fischer’s exact test. A p-value of less than 0.05 was considered significant.

## Results

The sociodemographic characteristics of the elderly participants and the factors affecting self-care are given in the form of tables. The Barthel index scoring and self-care ability of the participants are represented in the form of pie charts.

Table 1 shows the sociodemographic details of the participants. 167 (74.2%) people belonged to the age group of 60 to <75 years, and 127 (56.4%) were males. 67 (29%) of the participants were illiterate, and 114 (50.7%) were unemployed. Among the 225 participants, 167 (74.2%) were married and 50 (22.2%) were widowed. The majority, 196 (87.1%), of the participants were living with their families, and 114 (51%) were financially supported by their families. 25 (11.1%) of them were pensioners.

**Table 1:** Sociodemographic characteristics of the elderly

Sociodemographic variables		Frequency (n=225)	Percentage
Age	60 to <75 Years	167	74.2%
	≥ 75years	58	25.8%
Gender	Male	127	56.4%
	Female	98	43.6%
Education	Illiterate	67	29.8%
	Primary	38	16.9%
	Middle	20	8.9%
	High school	18	8%
	Graduate	42	18.7%
	Postgraduate	40	17.8%
Occupation	Unemployed	114	50.7%
	Unskilled	28	12.4%
	Clerical	38	16.9%
	Professional	45	20%
Monthly income	>19,575	46	20.4%
	9788-19574	20	8.9%
	7323-9787	3	1.3%
	4894-7322	55	24.4%
	2936-4893	74	32.9%
	980-2835	27	12%
Marital status	Married	167	74.2%
	Separated	8	3.6%
	Widow	50	22.2%
Living with	Family	196	87.1%
	Alone	29	12.9%
Source of income	Family members	114	50.7%
	Pension	25	11.1%
	Own	86	38.2%

**Figure 1:**
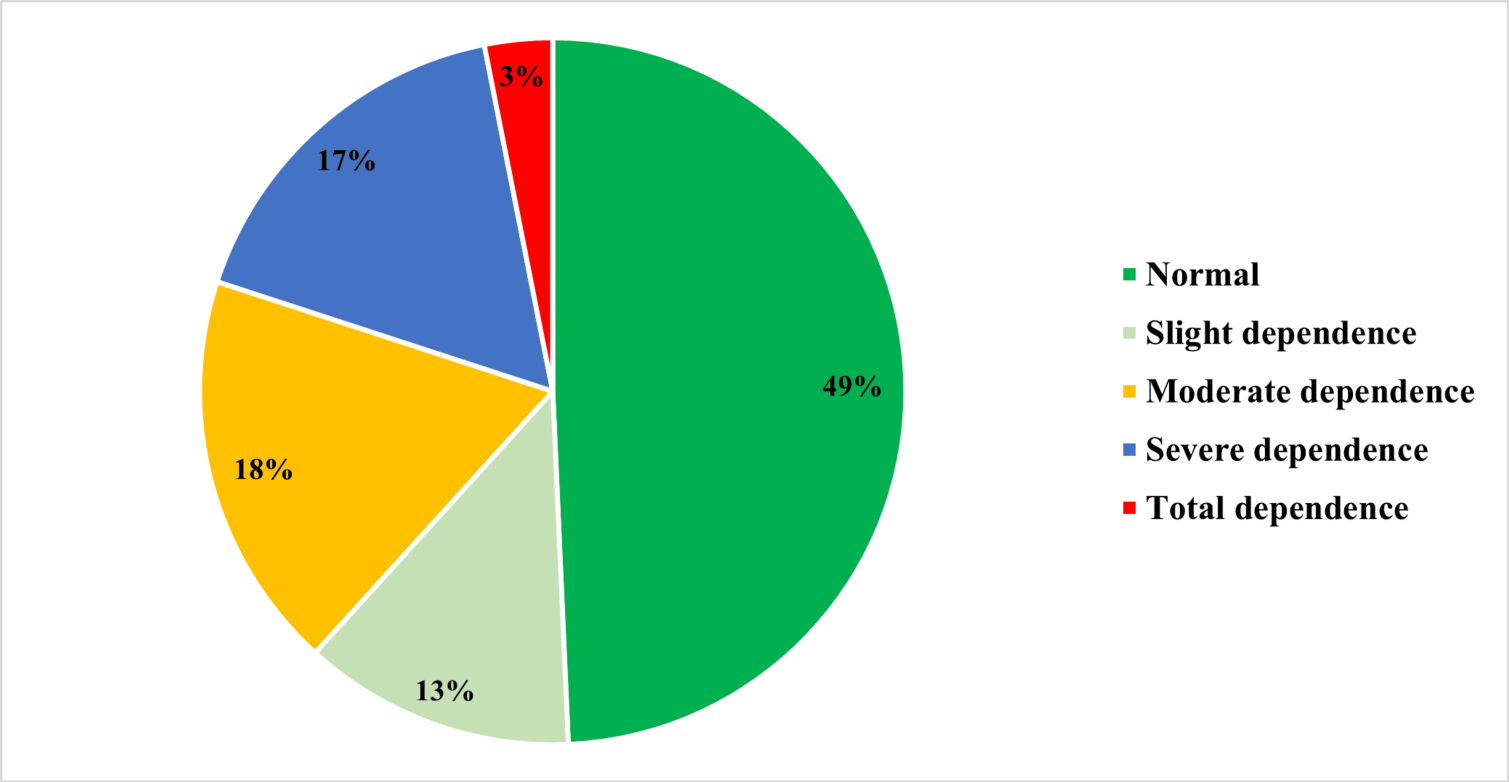
Barthel Index score

Figure 1 shows the Barthel Index score and the level of dependence among the elderly. Among the 225 participants, 41 of the elderly participants had moderate dependence, 38 were severely dependent, and 29 were slightly dependent. Seven of the elderly were totally dependent. 

**Figure 2:**
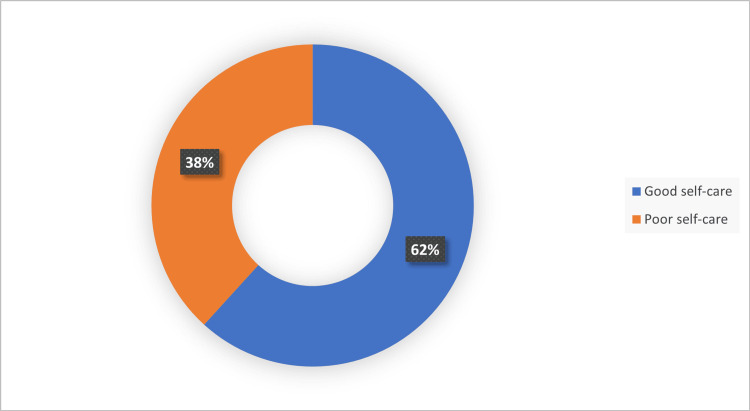
Self-care practices among the elderly

Figure 2 shows the self-care practices among the elderly in this study. Good self-care was practiced among 62% of the participants.

Table 2 shows the association between self-care and the factors affecting it.

**Table 2:** Factors affecting self-care and its association *p-value <0.05 is considered as statistically significant; H/O: history of; CAD: coronary artery disease

Variables	Category	Poor Self-care	Good Self-care	Chi-square (χ²)	P-value*	Odds ratio	95% CI
		Frequency (%)	Frequency (%)				
Age	≥ 75years	53 (91.4%)	5 (8.6%)	93.512	0.00*	43.04	15.97-116.174
	60 to <75 years	33 (19.8%)	134 (80.2%)				
Gender	Male	60 (47.2%)	67 (52.8%)	10.05	0.002*	0.40	0.229-0.712
	Female	26 (26.5%)	72 (73.5%)				
H/O Hypertension	Present	63 (40.9%)	91 (59.1%)	1.492	0.2	1.44	0.7-2.6
	Absent	23 (32.4%)	48 (67.6%)				
H/O Diabetes	Present	73 (51.8%)	68 (48.2%)	29.37	0.00*	5.86	2.97-11.54
	Absent	13 (15.5%)	71 (84.5%)				
H/O CAD	Present	28 (54.9%)	23 (45.1%)	7.77	0.005*	2.43	1.29-4.59
	Absent	58 (33.3%)	116 (66.7%)				
Duration of illness	< 5 years	39 (31.2%)	86 (68.8%)				
	>5 years	47 (47%)	53 (53%)	5.87	0.015*	1.95	1.13-3.37
Takes Medication on their own	Yes	41 (24.1%)	129 (75.9%)				
	No	45 (81.8%)	10 (18.2%)	58.59	0.000*	0.07	0.03-0.15
Routine follow-up	Yes	26 (20.8%)	99 (79.2%)				
	No	60 (60%)	40 (40%)	36.154	0.00*	0.17	0.097-0.315

Poor self-care was high among the elderly ≥75 years of age (23.5%) compared to those aged 60 to <75 years (14.7%). The association between age and self-care was found to be statistically significant (p <0.05). Poor self-care was observed among 47.6% of the males compared to 26.5% of females and this association was statistically significant (p <0.05).

History of diabetes and coronary artery disease in the elderly were statistically significant factors of poor self-care. Participants who had illnesses for more than 5 years and those who did not take medications on their own and those who were not on routine follow-up had poor self-care practices and the association was statistically significant.

## Discussion

In this study, the self-care practices of the elderly and the factors that contribute to the self-care practices were investigated. Since there was a lack of previous local studies, the study has addressed factors that were not thoroughly examined by other studies, such as taking prescribed medications on one's own, and offers additional information on the population in the area.

In the present study, around 62% of the elderly practiced good self-care. A similar finding of 55.9% of good self-care was reported by Getie et al [16] in their study done among adults in Eastern Ethiopia. Saraswat et al [13] found that good self-care was seen in half of their study participants (50.4%). Whereas poor self-care was seen among people aged more than 75 years old and elderly males in a study done by Safaeian et al [12]. Salehi et al [9] also observed that self-care was poor with increasing age and in men. Koirala et al documented a strong association between self-care and variables like age, gender, education, and health conditions. The desire for self-care decreased with growing age [17]. A study done by Gupta et al [14] and Murugan et al [18] showed an association between increasing age and poor self-care, the findings of which are similar to our study. This can be explained by the fact that with advancing age, there is often a decline in physical and cognitive functioning and an increased burden of comorbidities, all of which can hinder the ability to carry out regular self-care practices. Also, older adults may face social isolation, financial strain, and psychological issues, which further compromise their capacity for self-management.

In our study, factors such as history of diabetes and coronary artery disease were statistically significantly associated with poor self-care. Joshi et al [19] found that chronic health conditions significantly disturbed the daily activities of the elderly. Similar findings were seen by Chakrabarty et al in relation to ischemic heart disease and self-care [20]. Duration of illness >5 years was associated with poor self-care in the present study. A similar association was found in a study done in Turkey, which showed a decreasing self-care capacity as the disease duration increased in the study population [21].

A significant association was seen between taking prescribed medications by own and good self-care. This was a new finding of the study and can be explained by the reason that the ability to manage medications independently reflects higher functional status, better cognitive ability, and greater health awareness, all of which contribute towards effective self-care.

The present study found that those who were under routine follow-up had better self-care than those who were not. A similar finding was seen in a study done by Koç and Sağlam [22]. This may be explained by the reason that regular follow-up visits motivate self-care practices through patient education, timely identification, and management of complications. These interactions enhance health literacy, motivation, and adherence to self-care behaviors, ultimately leading to better disease management.

The Barthel index score in our study showed that 18% of the elderly were moderately dependent, 17% were severely dependent, and 3% were totally dependent. This is similar to the study by Gupta et al [14], where 23.4% of the participants were dependent on at least one activity of daily living. A study conducted in an urban area of Bengaluru reported that 37% of the elderly were totally dependent and 27% were mildly dependent [23]. Dale et al [24] documented that self-care was higher in older people living in rural areas, as they asked for more help from their families when compared to people in urban areas. A study done by Guigoz et al has also reported rural-urban differences in the prevalence of dependence in the elderly [25].

The major limitation of the study is that the causal relationship between self-care and its associated factors could not be established due to its descriptive nature. As this is self-reported data from the elderly, there may be a chance of recall bias, social desirability bias, and measurement error.

## Conclusions

The present study shows a higher prevalence of poor self-care among the elderly. The elderly age group is vulnerable to various disabilities, and their problems are often overlooked. Hence, good self-care and effective interventions must be of prime importance. Further analytical studies focusing on effective interventions will help to formulate policies to improve the quality of life of the geriatric community. Strengthening the implementation of Geriatric OPDs, domiciliary care, and caregiver education can significantly enhance self-care practices among the elderly.

Healthcare providers across all tiers of the health system must be trained routinely on the practices of self-care so that this knowledge can be imparted to every elderly person, especially patients suffering from non-communicable diseases, during each contact. At the level of the family, caregivers, spouses, and children of the patients all have a very important role in the elderly’s health and adherence to self-care behaviors. Hence, they should also be educated.

## Appendices

**Table 3:** General questionnaire

S. No.		
1)	Participant ID	
2)	Age	
	60 years to < 75 years	
	≥75 years	
3)	Gender	
	Male	
	Female	
4)	Educational qualification	
	Professions of honour / post graduate	
	Graduate / diploma	
	High school	
	Middle school	
	Primary school	
	Illiterate	
5)	Occupation	
	Professionals/Manager	
	Technicians/Skilled worker	
	Clerks /shopkeepers/market sales worker / semi -skilled worker	
	Unskilled worker	
	Unemployed	
6)	Marital Status	
	Never married	
	Married	
	Separated	
	Widowed	
7)	Living with	
	Family members	
	Alone	
8)	Income per month	
9)	Source of Income	
	Pension	
	Family members	
	Own	
10)	Do you have any health insurance?	
	Yes	
	No	
11	If yes, type of insurance	
	Government	
	Private	
12)	History of Non communicable diseases	
	Diabetes mellitus	
	Hypertension	
	Coronary artery disease	
	Cerebrovascular accident	
	Dyslipidaemia	
	Chronic obstructive lung dis	
	Cataract	
	Chronic kidney disease	
	Cancer	
13)	Duration of illness	
	<5 years	
	>5 years	
14)	Do you take the prescribed medications regularly?	
	Yes	
	No	
15)	Do you take the medications on your own?	
	Yes	
	No	
16	Do you go for routine follow-up?	
	Yes	
	No	
16)a	If yes, do you require any assistance?	
	Yes	
	No	

**Table 4:** Self-care questionnaire Source: Mahoney and Barthel [26]. Used with permission.

S. No.	Variables	Self-care scoring	Participant score
A.	Feeding	0 = unable 5 = needs help cutting, spreading butter, etc.or requires modified diet 10 = independent	
B.	Bathing	0 = dependent 5 = independent (or in shower)	
C	Grooming:	0 = needs to help with personal care 5 = independent face/hair/teeth/shaving (implements provided)	
D	Dressing	0 = dependent 5 = needs help but can do about half unaided 10 = independent (including buttons, zips, laces, etc.)	
E	Bowels	0 = incontinent (or needs to be given enemas) 5 = occasional accident 10 = continent	
F	Bladder	0 = incontinent, or catheterized and unable to manage alone 5 = occasional accident 10 = continent	
G	Toilet use	0 = dependent 5 = needs some help, but can do something alone 10 = independent (on and off, dressing, wiping)	
H	Transfers (bed to chair and back	0 = unable, no sitting balance 5 = major help (one or two people, physical), can sit 10 = minor help (verbal or physical) 15 = independent	
I	Mobility (on level surfaces)	0 = immobile or < 50 yards 5 = wheelchair independent, including corners, > 50 yards 10 = walks with help of one person (verbal or physical) > 50 yards 15 = independent (but may use any aid; for example, stick) > 50 yards	
J	Stairs	0 = unable 5 = needs help (verbal, physical, carrying aid) 10 = independent	

## References

[1] (2002). Ageing and health. https://www.who.int/news-room/fact-sheets/detail/ageing-and-health.

[2] (2002). Highlights From the 2024 World Population Data Sheet. https://www.prb.org/articles/highlights-from-the-2024-world-population-data-sheet/.

[3] Katz S, Stroud MW 3rd (1989). Functional assessment in geriatrics. A review of progress and directions. J Am Geriatr Soc.

[4] (2002). World Senior Citizen Day 2024 | Press Information Bureau. https://www.pib.gov.in/PressNoteDetails.aspx?NoteId=152034.

[5] (2024). Elderly in India 2021 | Ministry of Statistics and Programme Implementation, National Statistical Office, Government of India. https://mospi.gov.in/sites/default/files/publication_reports/Elderly%20in%20India%202021.pdf.

[6] (2024). 107th Issue of Sarvekshana - Journal of National Statistical Office | Government of India, Ministry of Statistics and Programme Implementation, National Statistical Office, New Delhi. https://mospi.gov.in/sites/default/files/publication_reports/107th%20issue%20of%20Sarvekshana%20Revised.pdf..

[7] Gijsen R, Hoeymans N, Schellevis FG, Ruwaard D, Satariano WA, van den Bos GA (2001). Causes and consequences of comorbidity: a review. J Clin Epidemiol.

[8] (2004). Self-care for health and well-being. https://www.who.int/news-room/fact-sheets/detail/self-care-health-interventions.

[9] Salehi L, Keikavoosi-Arani L (2020). Using the Backman model in determining the dimensions of self-care and its factors affecting the elderly in Tehran City, Iran. Intern Med Today.

[10] Železnik D (2007). Self-Care of the Home-Dwelling Elderly People Living in Slovenia. https://oulurepo.oulu.fi/handle/10024/37407.

[11] Backman K, Hentinen M (2001). Factors associated with the self-care of home-dwelling elderly. Scand J Caring Sci.

[12] (2002). ResearchGate [Internet]. [cited 2024 Dec 2]. (PDF) Self-care Ability and Demographic Characteristics among Older Adults in the Urban and Rural Areas of Miandoab, Iran. Available from. https://www.researchgate.net/publication/378902242_Self-care_Ability_and_Demographic_Characteristics_among_Older_Adults_in_the_Urban_and_Rural_Areas_of_Miandoab_Iran.

[13] Saraswat A, Eram U, Shah S, Ahmad A (2023). Study on assessment of self-care practices in patients of non communicable diseases in Aligarh. Ind J Pub Health Res.

[14] Gupta S, Yadav R, Malhotra AK (2016). Assessment of physical disability using Barthel index among elderly of rural areas of district Jhansi (U.P), India. J Family Med Prim Care.

[15] El-Osta A, Sasco ER, Barbanti E (2023). Tools for measuring individual self-care capability: a scoping review. BMC Public Health.

[16] Getie A, Geda B, Alemayhu T, Bante A, Aschalew Z, Wassihun B (2020). Self- care practices and associated factors among adult diabetic patients in public hospitals of Dire Dawa administration, Eastern Ethiopia. BMC Public Health.

[17] Koirala J, Raddi SA, Shivaswamy MS, Koirala D (2019). The knowledge and practices of self-care capabilities among geriatric population. Int J Biochem Physiol.

[18] Murugan Y, Parmar A, Hirani MM, Babaria DL, Damor NC (2024). Self-care practices and health-seeking behaviors among older adults in urban Indian slums: a mixed methods study. Cureus.

[19] Joshi K, Kumar R, Avasthi A (2003). Morbidity profile and its relationship with disability and psychological distress among elderly people in Northern India. Int J Epidemiol.

[20] Chakrabarty D, Mandal PK, Manna N (2010). Functional disability and associated chronic conditions among geriatric populations in a rural community of India. Ghana Med J.

[21] Tel H, Tel H, Sabancıoğulları S (2006). Status of maintenance of activities of daily living and experience of loneliness in elder than 60 years old living at home and in institutions [Evde ve kurumda yaşayan 60 yaş ve üzeri bireylerin günlük yaşam aktivitelerini sürdürme ve yalnızlık yaşama durumu]. Turk Geriatri Dergisi.

[22] Koç Z, Sağlam Z (2019). Determination of the effects of daily life activities and self-care capacity on depression of the elderly in Northern Turkey. Acta Clin Croat.

[23] Srinivasan K, Vaz M, Thomas T (2010). Prevalence of health related disability among community dwelling urban elderly from middle socioeconomic strata in Bangaluru, India. Indian J Med Res.

[24] Dale B, Saevareid HI, Kirkevold M, Söderhamn O (2010). Older home nursing patients' perception of social provisions and received care. Scand J Caring Sci.

[25] Guigoz Y, Vellas B, Garry PJ (1996). Assessing the nutritional status of the elderly: the Mini Nutritional Assessment as part of the geriatric evaluation. Nutr Rev.

[26] Mahoney FI, Barthel DW (1965). Functional evaluation: the Barthel index. Md State Med J.

